# Supplementary material for: A multi-institutional study using artificial intelligence to provide reliable and fair feedback to surgeons
Source: Commun Med (Lond). 2023 Mar 30;3:42. doi: 10.1038/s43856-023-00263-3 (PMC10063640; doi:10.1038/s43856-023-00263-3)
Supplement: Supplementary file 6 — Description of Additional Supplementary Files [file 43856_2023_263_MOESM6_ESM.pdf]

Description of Additional Supplementary Files

File Name: Supplementary Data 1

Description: Heatmap of the ground-truth explanation annotations for video samples of needle handling and needle driving across hospitals.

File Name: Supplementary Data 2

Description: Precision-recall curves reflecting the alignment of different AI-based explanations with those provided by humans when assessing the skill-level of needle handling and needle driving.

File Name: Supplementary Data 3

Description: Reliability of attention-based explanations stratified across surgeon sub-cohorts when assessing the skill-level of needle handling and needle driving.

File Name: Supplementary Data 4

Description: Effect of TWIX on the reliability of AI-based explanations for the disadvantaged surgeon sub-cohort (worst-case AUPRC) when assessing the skill-level of needle handling and needle driving.

File Name: Supplementary Data 5

Description: Ablation studies and effect of TWIX when SAIS is deployed on data from a training environment.
