# Supplementary material for: A multi-institutional study using artificial intelligence to provide reliable and fair feedback to surgeons
Source: Commun Med (Lond). 2023 Mar 30;3:42. doi: 10.1038/s43856-023-00263-3 (PMC10063640; doi:10.1038/s43856-023-00263-3)
Supplement: Supplementary file 7 — Supplementary Information [file 43856_2023_263_MOESM7_ESM.pdf]

# Supplementary Information

# A multi-institutional study using artificial intelligence to provide reliable and fair feedback to surgeons

**Dani Kiyasseh[1,*], Jasper Laca[2], Taseen F. Haque[2], Brian J. Miles[3], Christian Wagner[4], Daniel A. Donoho[5], Animashree Anandkumar[1], and Andrew J. Hung[2,*]**

[1]Department of Computing and Mathematical Sciences, California Institute of Technology, CA, USA
[2]Center for Robotic Simulation and Education, Catherine & Joseph Aresty Department of Urology, University of Southern California, CA, USA
[3]Department of Urology, Houston Methodist Hospital, TX, USA
[4]Department of Urology, Pediatric Urology and Uro-Oncology, Prostate Center Northwest, St. Antonius-Hospital, Gronau, Germany
[5]Division of Neurosurgery, Center for Neuroscience, Children's National Hospital, Washington DC, USA
[*]Corresponding authors: danikiy@hotmail.com, andrew.hung@med.usc.edu

| | needle handling | | | needle driving | | |
|---|---|---|---|---|---|---|
| | USC | SAH | HMH | USC | SAH | HMH |
| video samples | 26 | 8 | 11 | 27.5 | 7 | 14 |
| video explanations | 8 | 3 | 4 | 8 | 3 | 4 |
| explanation percentage % | 31 | 38 | 36 | 29 | 43 | 29 |

**Supplementary Table 1. Average duration (seconds) of video samples and explanations.** We present the average duration of video samples and explanations of the needle handling and needle driving surgical activities across hospitals. The percentage reflects the proportion of the video sample which is deemed as important by the trained human raters.

| Fold | train | | | validation | | | test | | |
|---|---|---|---|---|---|---|---|---|---|
| | n | v | s | n | v | s | n | v | s |
| 0 | 748 | 63 | 17 | 82 | 7 | 6 | 82 | 8 | 4 |
| 1 | 752 | 63 | 18 | 82 | 7 | 5 | 78 | 8 | 6 |
| 2 | 778 | 63 | 16 | 44 | 7 | 6 | 90 | 8 | 6 |
| 3 | 730 | 63 | 18 | 102 | 7 | 6 | 80 | 8 | 6 |
| 4 | 728 | 63 | 17 | 60 | 7 | 5 | 124 | 8 | 7 |
| 5 | 774 | 63 | 16 | 46 | 7 | 6 | 92 | 8 | 6 |
| 6 | 724 | 63 | 16 | 102 | 7 | 6 | 86 | 8 | 8 |
| 7 | 752 | 63 | 16 | 102 | 7 | 5 | 58 | 8 | 6 |
| 8 | 754 | 63 | 19 | 86 | 7 | 6 | 72 | 8 | 5 |
| 9 | 756 | 63 | 17 | 90 | 7 | 4 | 66 | 8 | 6 |

| Fold | train | | | validation | | | test | | |
|---|---|---|---|---|---|---|---|---|---|
| | n | v | s | n | v | s | n | v | s |
| 0 | 442 | 63 | 17 | 42 | 7 | 6 | 46 | 8 | 4 |
| 1 | 438 | 63 | 18 | 42 | 7 | 5 | 50 | 8 | 6 |
| 2 | 432 | 63 | 16 | 44 | 7 | 6 | 54 | 8 | 6 |
| 3 | 452 | 63 | 18 | 42 | 7 | 6 | 36 | 8 | 6 |
| 4 | 438 | 62 | 17 | 38 | 7 | 5 | 54 | 8 | 7 |
| 5 | 448 | 63 | 16 | 30 | 7 | 6 | 52 | 8 | 6 |
| 6 | 400 | 63 | 16 | 62 | 7 | 6 | 68 | 8 | 8 |
| 7 | 450 | 63 | 16 | 54 | 7 | 5 | 26 | 8 | 6 |
| 8 | 408 | 63 | 19 | 48 | 7 | 6 | 74 | 8 | 5 |
| 9 | 412 | 63 | 17 | 58 | 7 | 4 | 60 | 8 | 6 |

**Supplementary Table 2. Number of video samples (n), unique surgical videos (v), and surgeons (s) in each fold and data split at USC.** We used these samples in the 10-fold Monte Carlo cross-validation setup to train and evaluate SAIS in assessing the skill-level of needle handling (left) and needle driving (right).

| | caseload | | | | | | prostate volume | | | | | | Gleason score | | | | | | | | |
|---|---|---|---|---|---|---|---|---|---|---|---|---|---|---|---|---|---|---|---|---|---|
| | novice | | | expert | | | $\leq$ 49ml | | | $>$ 49ml | | | 6 | | | 7 | | | 8 | | |
| Fold | n | v | s | n | v | s | n | v | s | n | v | s | n | v | s | n | v | s | n | v | s |
| 0 | 14 | 3 | 3 | 25 | 6 | 3 | 10 | 2 | 2 | 31 | 6 | 5 | 14 | 2 | 3 | 9 | 3 | 4 | 18 | 3 | 2 |
| 1 | 17 | 3 | 3 | 21 | 5 | 4 | 17 | 5 | 5 | 22 | 3 | 4 | 16 | 3 | 4 | 17 | 4 | 5 | 6 | 1 | 1 |
| 2 | 15 | 3 | 3 | 22 | 6 | 5 | 22 | 4 | 4 | 23 | 4 | 6 | 18 | 2 | 2 | 14 | 4 | 5 | 8 | 1 | 1 |
| 3 | 31 | 6 | 5 | 8 | 3 | 3 | 30 | 6 | 6 | 10 | 2 | 4 | 13 | 3 | 3 | 23 | 4 | 6 | 4 | 1 | 1 |
| 4 | 33 | 5 | 4 | 21 | 3 | 2 | 24 | 4 | 4 | 38 | 4 | 4 | 20 | 3 | 3 | 34 | 4 | 4 | 8 | 1 | 1 |
| 5 | 4 | 1 | 1 | 40 | 6 | 6 | 23 | 3 | 3 | 12 | 4 | 4 | 2 | 1 | 1 | 32 | 5 | 5 | 12 | 2 | 2 |
| 6 | 27 | 4 | 4 | 14 | 5 | 5 | 8 | 3 | 3 | 35 | 5 | 7 | 3 | 2 | 2 | 35 | 4 | 6 | 5 | 2 | 2 |
| 7 | 7 | 2 | 2 | 20 | 5 | 5 | 12 | 4 | 4 | 17 | 4 | 4 | 14 | 3 | 3 | 15 | 5 | 5 | | | |
| 8 | 16 | 2 | 2 | 20 | 7 | 7 | 22 | 4 | 5 | 14 | 4 | 4 | | | | 21 | 4 | 5 | 15 | 4 | 4 |
| 9 | 13 | 3 | 3 | 17 | 6 | 4 | 7 | 2 | 3 | 26 | 6 | 6 | 10 | 1 | 2 | 15 | 5 | 6 | 3 | 1 | 1 |

**Supplementary Table 3. Number of video samples (n), unique surgical videos (v), and surgeons (s) in each test fold across surgeons groups when assessing the skill-level of needle handling.** We used these samples when stratifying the reliability of explanations across surgeon groups.

| | caseload | | | | | | prostate volume | | | | | | Gleason score | | | | | | | | |
|---|---|---|---|---|---|---|---|---|---|---|---|---|---|---|---|---|---|---|---|---|---|
| | novice | | | expert | | | $\leq$ 49ml | | | $>$ 49ml | | | 6 | | | 7 | | | 8 | | |
| Fold | n | v | s | n | v | s | n | v | s | n | v | s | n | v | s | n | v | s | n | v | s |
| 0 | 5 | 3 | 3 | 18 | 6 | 3 | 6 | 1 | 1 | 17 | 6 | 5 | 6 | 2 | 3 | 5 | 2 | 3 | 12 | 3 | 2 |
| 1 | 5 | 2 | 2 | 19 | 5 | 5 | 11 | 4 | 5 | 14 | 3 | 4 | 10 | 3 | 4 | 10 | 3 | 5 | 5 | 1 | 1 |
| 2 | 10 | 2 | 2 | 15 | 6 | 5 | 14 | 4 | 4 | 13 | 4 | 5 | 6 | 2 | 2 | 16 | 4 | 4 | 2 | 1 | 1 |
| 3 | 13 | 3 | 3 | 4 | 3 | 3 | 10 | 4 | 4 | 8 | 2 | 3 | 5 | 2 | 2 | 11 | 3 | 4 | 2 | 1 | 1 |
| 4 | 16 | 4 | 4 | 9 | 3 | 2 | 8 | 3 | 3 | 19 | 4 | 4 | 6 | 2 | 2 | 19 | 4 | 4 | 2 | 1 | 1 |
| 5 | 4 | 1 | 1 | 22 | 7 | 7 | 15 | 3 | 4 | 7 | 3 | 3 | | | | 19 | 5 | 6 | 7 | 2 | 2 |
| 6 | 21 | 3 | 3 | 13 | 5 | 5 | 10 | 3 | 3 | 24 | 4 | 5 | 1 | 1 | 1 | 22 | 4 | 5 | 11 | 2 | 2 |
| 7 | 1 | 1 | 1 | 12 | 4 | 4 | 6 | 2 | 2 | 7 | 3 | 3 | 6 | 3 | 3 | 7 | 2 | 2 | | | |
| 8 | 9 | 2 | 2 | 28 | 6 | 6 | 20 | 4 | 6 | 17 | 4 | 3 | | | | 15 | 4 | 4 | 22 | 4 | 4 |
| 9 | 7 | 2 | 2 | 19 | 7 | 5 | 9 | 2 | 4 | 21 | 6 | 5 | 5 | 1 | 2 | 15 | 5 | 6 | 7 | 1 | 1 |

**Supplementary Table 4. Number of video samples (n), unique surgical videos (v), and surgeons (s) in each test fold across surgeons groups when assessing the skill-level of needle driving at USC.** We used these samples when stratifying the reliability of explanations across surgeon groups.

| | prostate volume | | | | | | Gleason score | | | | | | | | | | | |
|---|---|---|---|---|---|---|---|---|---|---|---|---|---|---|---|---|---|---|
| | ≤ 49ml | | | > 49ml | | | 6 | | | 7 | | | 8 | | | 9 | | |
| | n | v | s | n | v | s | n | v | s | n | v | s | n | v | s | n | v | s |
| handling | 81 | 18 | 5 | 39 | 9 | 4 | 23 | 7 | 4 | 66 | 13 | 5 | 18 | 5 | 3 | 13 | 2 | 2 |
| driving | 88 | 18 | 5 | 52 | 9 | 4 | 49 | 7 | 4 | 67 | 13 | 5 | 17 | 5 | 3 | 7 | 2 | 2 |

**Supplementary Table 5. Number of video samples in each surgeon group from St. Antonius Hospital.** We used these video samples to stratify the reliability of explanations (whether attention-based or TWIX) across surgeon sub-cohorts.

| | caseload | | | | | | prostate volume | | | | | | Gleason score | | | | | | | | | | | |
|---|---|---|---|---|---|---|---|---|---|---|---|---|---|---|---|---|---|---|---|---|---|---|---|---|
| | novice | | | expert | | | ≤ 49ml | | | > 49ml | | | 6 | | | 7 | | | 8 | | | 9 | | |
| | n | v | s | n | v | s | n | v | s | n | v | s | n | v | s | n | v | s | n | v | s | n | v | s |
| handling | 36 | 10 | 1 | 49 | 10 | 3 | 53 | 13 | 3 | 16 | 3 | 2 | 2 | 1 | 1 | 61 | 14 | 4 | 17 | 4 | 2 | 5 | 1 | 1 |
| driving | 57 | 10 | 1 | 46 | 10 | 3 | 71 | 13 | 3 | 16 | 3 | 2 | 4 | 1 | 1 | 61 | 14 | 4 | 31 | 4 | 2 | 7 | 1 | 1 |

**Supplementary Table 6. Number of video samples in each surgeon group from Houston Methodist Hospital.** We used these video samples to stratify the reliability of explanations (whether attention-based or TWIX) across surgeon sub-cohorts.

| | gender | | | | | |
|---|---|---|---|---|---|---|
| | male | | | female | | |
| | n | v | s | n | v | s |
| handling | 64 | 29 | 17 | 100 | 40 | 21 |

**Supplementary Table 7. Number of video samples in each group from the laboratory environment.** We used these video samples to stratify the reliability of explanations (whether attention-based or TWIX) across sub-cohorts.

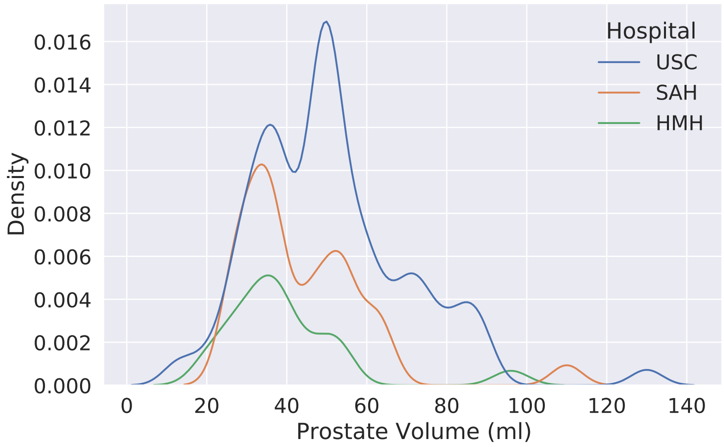


**Supplementary Figure 1. Distribution of prostate volume of patients across the three hospitals.** These distributions are based on the total number of video samples from each hospital. Note the difference in the average prostate volume across hospitals.
